# Investigating diarrhoea on the ICU: a retrospective study

**DOI:** 10.1186/cc10766

**Published:** 2012-03-20

**Authors:** N Tirlapur, M Kelsey, H Montgomery

**Affiliations:** 1 Whittington Hospital, London, UK

## Introduction

Diarrhoea is common in ICU patients, with a reported prevalence of 15 to 38% [1]. Many factors may cause diarrhoea, including *Clostridium difficile*, drugs (for example, laxatives, antibiotics), faecal impaction with overflow and enteral feeds. Diarrhoea increases nursing workload, impacts on patient dignity, increases costs and exacerbates morbidity through dermal injury, impaired enteral uptake and subsequent fluid imbalance. We aimed to identify prevalence, yield of stool investigations and clinical impact of diarrhoea on our ICU.

## Methods

A retrospective observational study of all ICU patients treated in a 15-bed district general hospital from 1 January 2010 to 31 December 2010 was performed. ICU patients from whom stool samples had been sent for microbiological analysis (including microscopy and *C. difficile *toxin (CDT)) were assumed to have suffered diarrhoea. Stool sample results were compiled with patient demographics, ICU length of stay (LOS) and mortality data.

## Results

Of 782 patients (mean age ± 2SD 62.1 ± 37.1, 52.3% female) treated on our ICU, 334 stool samples were sent from 133 (17.0%) patients. Two samples (0.6%) yielded abnormal results: one out of 131 (0.8%) patients with CDT samples sent and one out of 108 (0.9%) patients with stool microscopy samples sent had a positive sample. The prevalence of *C. difficile *(1/782) and other organisms (1/782) was 0.1% and 0.1% respectively. In terms of diagnostic yields, positive findings were found in one out of 191 (0.5%) CDT samples and one out of 141 (0.7%) stool microscopy samples (for *Candida*). When compared to patients without diarrhoea, sufferers were older (64.1 ± 33.2 vs. 61.7 ± 37.8 years, *P *= 0.16) with greater female preponderance (55.6% vs. 51.6%, *P *= 0.40). Sufferers experienced longer ICU LOS (16.3 ± 45.6 vs. 4.6 ± 19.4 days, *P *< 0.0001) and greater ICU mortality (19.5% vs. 12.6%, *P *= 0.035) during the study period.

## Conclusion

Diarrhoea was common on our ICU, its prevalence (17%) being consistent with established literature. It was associated with statistically increased ICU LOS and mortality, although any direction of causality remains to be established. A low stool investigation yield and low prevalence of *C. difficile *suggests that other noninfective causes of diarrhoea need excluding. Further research is required to establish the prevalence and pathogenesis of diarrhoea on UK ICUs, in order to develop evidence-based management plans for reducing its incidence, and its clinical and financial impact.
